# *KYNU*, a novel potential target that underpins CD44‐promoted breast tumour cell invasion

**DOI:** 10.1111/jcmm.16296

**Published:** 2021-01-24

**Authors:** Maryam Al‐Mansoob, Ishita Gupta, Radoslaw Stefan Rusyniak, Allal Ouhtit

**Affiliations:** ^1^ Department of Biological & Environmental Sciences College of Arts and Science Qatar University Doha Qatar; ^2^ College of Medicine QU Health Qatar University Doha Qatar

**Keywords:** breast cancer, CD44, Hyaluronan, *KYNU*

## Abstract

Using a validated tetracycline‐off‐inducible CD44 expression system in mouse model, we have previously demonstrated that the hyaluronan (HA) receptor CD44 promotes breast cancer (BC) metastasis to the liver. To unravel the mechanisms that underpin CD44‐promoted BC cell invasion, RNA samples were isolated from two cell models: (a) a tetracycline (Tet)‐Off‐regulated expression system of the CD44s in MCF‐7 cells and; (b) as a complementary approach, the highly metastatic BC cells, MDA‐MB‐231, were cultured in the presence and absence of 50 µg/mL of HA. Kynureninase (*KYNU*), identified by Microarray analysis, was up‐regulated by 3‐fold upon induction and activation of CD44 by HA; this finding suggests that *KYNU* is a potential novel transcriptional target of CD44‐downtstream signalling. KYNU is a pyridoxal phosphate (PLP) dependent enzyme involved in the biosynthesis of NAD cofactors from tryptophan that has been associated with the onset and development of BC. This review will attempt to identify and discuss the findings supporting this hypothesis and the mechanisms linking *KYNU* cell invasion *via* CD44.

**Abbreviations**
3‐HAA: anthranilic acid and 3‐hydroxyanthranilic acid
3HK: 3‐hydroxy‐L‐kynurenine
AKT: Protein kinase B
BC: Breast cancer
CAM: Cell adhesion molecule
CD44: Cluster of differentiation 44
cSCC: Cutaneous squamous cell carcinoma
HA: Hyaluronan
KMO: Kynurenine 3‐monooxygenase
KYNU: Kynureninase
NAD: Nicotinamide adenine dinucleotide
NAH: N‐acetylhistidine
NF‐κB: Nuclear factor‐kappa beta
PI3K: phosphoinositide 3‐kinase
PLP: pyridoxal‐5’‐phosphate
QUIN: Quinolinic acid
Ras: Rat sarcoma
Tet: Tetracycline
TGF‐β2: Transforming growth factor‐beta 2
TNF: Tumour necrosis factor
VCRL2: Vertebral, cardiac, renal and limb defects syndrome 2
XA: xanthurenic acid

## 1 INTRODUCTION

Breast cancer (BC) is the most commonly diagnosed malignancy in women worldwide, including the Middle East region and Qatar, accounting for around 1/4th of all cancer cases.^1^ BC cells frequently metastasize to the bone, liver, lung and brain,^2^ and it is this ability of tumour cells to detach from the primary tumour, migrate and invade a new location in the body that is the most devastating aspect of cancer.^3^ This process relies on cell adhesion molecules (CAM), located on cell surfaces for their essential role in cell‐to‐cell and cell‐extracellular adhesion.^4^ CAMs form several protein families including cadherins, integrins, selectins and immunoglobins.^4^, ^5^

CD44 is an adhesion protein belonging to the CAM family and is the primary receptor for its ligand, Hyaluronic acid (HA) involved in regulating cellular proliferation, migration and invasion signalling.^6^ In order to understand the function and signalling pathways involved in CD44‐mediated tumour cell invasion and metastasis, we developed a tetracycline (Tet)‐Off‐regulated expression system of the CD44s both in vitro,^7^ and *in* *vivo* ^8^ and performed microarray analysis (12K CHIP from Affymetrix) to identify genes under the direct regulation of CD44‐downstream signalling. From the obtained list of targets, we have previously validated three genes (*Cortactin,* *Survivin* *and* *TGF‐β2*) as target genes that underpin CD44, along with their downstream signalling pathways.^7^, ^8^, ^9^ Among these 200 genes, we have selected *Kynureninase* (*KYNU*) in order to provide and discuss lines of evidence from the literature, supporting our hypothesis that *KYNU* might be a novel transcriptional target of CD44‐downstream signalling.

**FIGURE 1:**
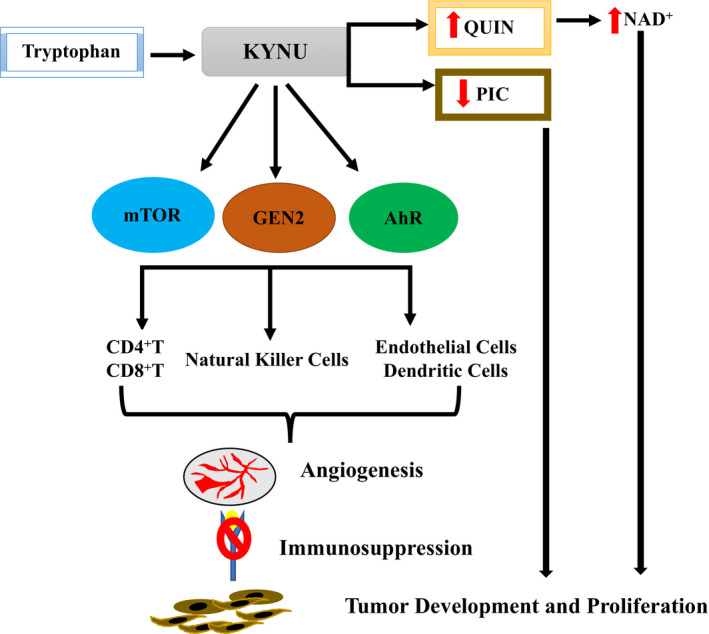
*KYNU* pathway involvement in mechanisms associated with tumour development

KYNU is a hydrolase involved in Tryptophan metabolism, contributing to the synthesis of NAD^+^ cofactors *via* the Kynurenine pathway; a vital pathway of L‐tryptophan catabolism in both bacteria as well as eukaryotes.^10^ In the pathway, KYNU catalyses L‐kynurenine (bacteria) and 3‐hydroxy‐L‐kynurenine (3HK) (eukaryotes) through a pyridoxal‐5′‐phosphate (PLP) dependent mechanism, to produce anthranilic acid and 3‐hydroxyanthranilic acid (3‐HAA), respectively.^10^ KYNU is expressed in almost all body organs, and in higher levels in the liver, the urinary bladder and the appendix.^11^ *KYNU* is involved in various inflammatory and cardiovascular diseases, in addition to several types of cancers, acting *via* different pathways (Figure 1).^12^, ^13^, ^14^, ^15^

Here we discuss the findings, from the literature, supporting the hypothesis that *KYNU* is a transcriptional target of CD44 as well as known signalling pathways linking the activation of CD44 by HA to the transactivation of KYNU in promoting breast tumour cell invasion.

## 2 STRUCTURE OF *KYNU*

*KYNU*, a member of the aminotransferase superfamily is located on the long arm of chromosome 2 (2q22; from base pairs 143 506 498 to base pairs 143 799 892), encompassing 293.39 kb of DNA, including 44 introns and 21 exons.^16^ *KYNU*, normally confined within the cytoplasm of the cells of various body tissues, requires the cofactor pyridoxal‐5′‐phosphate for its activity.^17^

KYNU protein consists of 465 amino acids and exists as a homodimer structurally homologous to other members of the PLP‐dependent aspartate aminotransferase family.^18^ Each monomer is composed of two regions: a small and a large domain, with a sizeable opening, containing the active site, formed at the junction between these domains in the dimerized form.^18^ Like other members of this family, KYNU’s active site features a conserved lysine, which forms the PLP‐enzyme Schiff base, with a variety of nearby amino acids, maintaining the cofactor's proper orientation through hydrogen bonding.^18^ Similarly, a conserved arginine appears to be critical to binding and orienting the substrate within the active site.^16^ This binding results in a conformational change that puts strain on the substrate's bonds, which would be released upon hydrolysis.^16^

## 3 FUNCTIONS OF *KYNU*

*KYNU* is involved in the biosynthesis of NAD + from tryptophan *via* the kynurenine pathway.^19^ Specifically, it degrades kynurenine, a catabolite in tryptophan metabolism, into anthranilic acid.

### 3.1 Physiological functions of *KYNU* in normal cells

In most mammalian cells, the KYNU pathway is the primary path of tryptophan metabolism, producing metabolites, such as kynurenic acid (KYNA), xanthurenic acid (XA), and 3‐hydroxyanthranilic acid (3‐HAA).^20^ Of these three, 3‐HAA is the main product of this pathway and is eventually converted to NAD+ (21), while KYNA and XA appear to only be produced when KYNU is fully saturated.^21^

### 3.2 Functions of *KYNU* in vertebral, cardiac, renal and limb defects syndrome 2 (VCRL2)

KYNU is linked with tryptophan utilization and metabolic diseases, including vertebral, cardiac, renal and limb defects syndrome 2 (VCRL2),^19^ which is an autosomal recessive congenital malformation syndrome; This association was made through a study of individuals with truncated *KYNU* genes; in vitro functional assays demonstrated that these mutations inhibited KYNU enzymatic activity, which resulted in enhanced 3HK levels and loss of NAD and NAH(H).^19^

Further, in vivo study demonstrated that heterozygous *KYNU* ^±^ mice with a niacin‐free diet during the early embryonic stages was able to sustain normal embryonic development due to the sufficient supply of the de novo NAD+.^19^ On the other hand, homozygous null *KYNU* ^−^/^−^ mice with a niacin‐free diet were inviable and all embryos died even when niacin was only limited from embryonic day 0.5 to 5. Further research in mutant *KYNU* ^−^/^−^ mice born to mothers on a niacin‐free diet demonstrated lack of NAD due to *KYNU* loss; mice developed several congenital abnormalities including cleft palate, club foot, syndactyly, and caudal regression syndrome.^19^ It was found that the elevated levels of niacin in mice were plausibly transferred from their mothers, thus, providing a protective effect on genetic‐based NAD paucity.^19^ To sum up, the kynurenine pathway synthesis of NAD + is essential and mutations in *KYNU* leads to congenital malfunctions and inviable embryos.^19^

### 3.3 Functions of *KYNU* in breast cancer and its association to CD44‐signalling

Although *KYNU* is often associated with metabolic diseases, its role in cancer lies nascent and only a few studies have investigated the link between *KYNU* and CD44, and its association with cancer. One of the key pathways dysregulated in cancer is the PI3K/AKT pathway; this pathway regulates various physiological functions, including cellular migration, invasion and cell survival. ^22^, ^23^ CD44 activates PI3K/AKT and Ras pathways through its interaction with HER‐2 and c‐Met, respectively.^7^, ^24^ The activation of this pathway has, however, been shown to be impacted by the knockdown of *KYNU,* which reduced levels of phosphorylated metabolites p‐PI3K/p‐AKT,^12^ and indicated a role for *KYNU* in triggering the PI3K/AKT pathway. It could be speculated that *KYNU* may be a plausible target of CD44 through which it interacts with HER‐2 and activates the downstream pathway. Thus, *KYNU* can be a candidate therapeutic target as the PI3K/AKT pathway can be modulated by silencing *KYNU*.

Another study analysing inflammation‐associated mechanisms, which are regulating and blocking the TNF‐induced signal transduction in primary human monocytes, identified *KYNU* as one of the proteins linked to nuclear factor κB (NF‐κB) pathway.^25^ Moreover, NF‐κB plays a crucial role in tumour invasion and metastasis,^26^ thus suggesting a role of *KYNU* in promoting tumour cell invasion. The study also reported that long‐term incubation of cells in TNF correlated with increased expression of *KYNU* and increased phosphorylation of CD44.^25^ Similarly, to the dual controversial role of many other genes (eg *p53*), while, some studies support its oncogenic role,^27^, ^28^, ^29^, ^30^, ^31^, ^32^, ^33^ a few studies have also shown its role as a tumour suppressor.^14^, ^34^

The first study by Rose et al, (1967) reported increased activity of the kynurenine pathway, along with increased *KYNU*, KMO and kynurenine aminotransferase‐II activity in BC patients,^33^ indicating an oncogenic role of *KYNU*. Further, microarray data from invasive BC patients showed differential expression of *KYNU* in the BC subtypes.^28^, ^30^ While, no change in *KYNU* expression was observed in the luminal subtypes, *KYNU* expression was enhanced in the HER2‐positive, claudin low and aggressive basal BC subtypes,^28^, ^30^, ^31^ thus linking *KYNU* overexpression with higher rates of lymph node metastasis and promotion of BC progression and metastasis. Interestingly, a study in cutaneous squamous cell carcinoma (cSCC) analysed the expression of *KYNU*, using Gene Expression Omnibus and the Oncomine databases, and further validated the results using structural and functional assays.^12^ The results showed that *KYNU* expression was up‐regulated in cSCC, and blocking *KYNU* significantly inhibited cSCC cell proliferation, migration and invasion, thus pointing to *KYNU* as a plausible target for therapeutic interventions for cSCC.^12^

In addition to our previous study showing CD44 activation of NF‐kB, another study revealed that NF‐kB induced significant expression of *KYNU* in triple‐negative BC cell lines, further supporting our hypothesis that CD44‐activated NF‐kB might transactivate *KYNU*.^29^ Such enhanced *KYNU* expression has been correlated with BC metastasis to the lung and brain.^27^, ^32^ On the other hand, RAS, the upstream pathway for PI3K/AKT pathway is associated with BC metastasis^35^; stimulating the RAS signalling pathway correlated with up‐regulation of the expression of KYNU,^36^ thus indicating an oncogenic role of *KYNU* in BC metastasis.

On the other hand, a recent study reported a negative correlation of *KYNU* expression and BC histological grades tumour stages; *KYNU* expression was lost in poorly differentiated BC cells and stage 3 BC.^14^ *KYNU* was negatively associated with HER‐2 expression and Ki‐67.^14^ Moreover, both in vitro and in vivo experiments revealed that loss of *KYNU* activated BC cell proliferation, differentiation, colony formation and xenograft tumour growth.^14^

## 4 POTENTIAL INHIBITORS OF *KYNU*

*KYNU* may play a role in underlying mechanisms resulting in the production of the excitotoxin moiety quinolinic acid (QUIN), which is a metabolite of tryptophan that has been shown to be a neurotoxin.^37^ Several studies have been carried out to develop suitable inhibitors targeting *KYNU* in order to guide the design of appropriate therapeutic strategies in bacteria as well as mammals. In fact, few of the bacterial KYNU inhibitors that mimic the molecular transition state of a range of substrates have been developed including (4S)‐ and (4R)‐dihydro‐L‐kynurenine,^38^ a series of S‐aryl‐L‐cysteine S,S‐dioxides^39^ and a phosphinic acid ^40^ analogue of kynurenine. On the other hand, in human macrophages, S‐aryl‐L‐cysteine S,S‐dioxides produced a molecule (2‐amino‐5‐methyl‐S‐phenyl cysteine S,S‐dioxide), which significantly blocked INF‐γ‐induced QUIN synthesis.^37^ Another kynurenine derivative (2‐amino‐4‐[3’‐hydroxy‐phenyl]‐4‐hydroxybutanoic acid) was developed in order to incorporate a hydroxy‐l moiety at C7 and C3 to resemble the substrates conversion state and enhance specificity towards human *KYNU*.^41^

## 5 CONCLUSION

**FIGURE 2:**
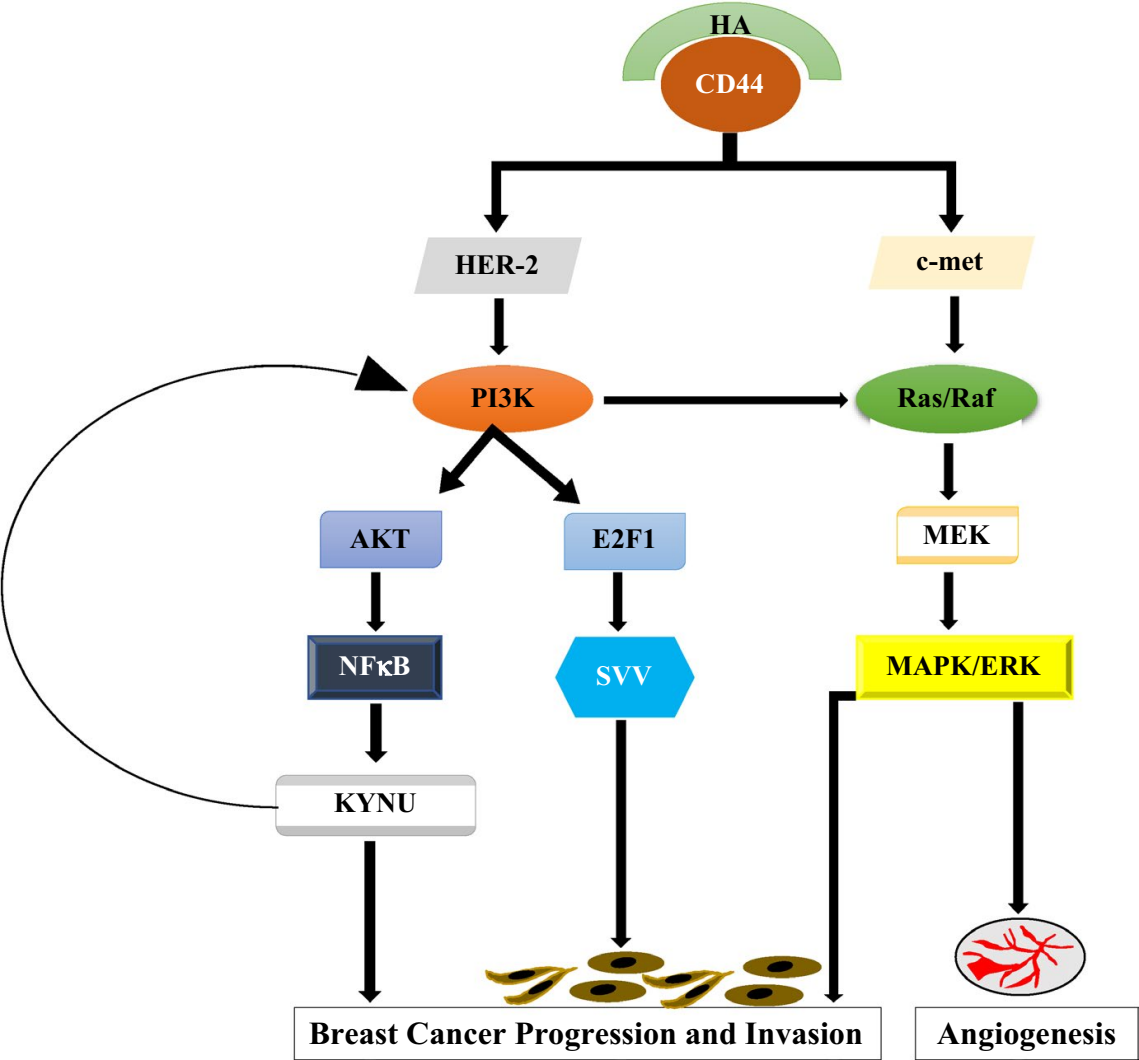
Suggested mechanisms linking HA‐CD44 interaction to *KYNU* transcription

*KYNU* appears to play a major role in the development and dissemination of breast tumours, but its underlying mechanisms are still poorly understood. *KYNU* interacts with several signalling pathways that promote breast tumour cell invasion and metastasis. In particular, findings from our own work and others support our hypothesis that CD44‐HA interaction might activate NF‐kB, which in turn transactivate *KYNU*, ultimately leading to BC cell invasion (Figure 2).

On the other hand, another finding from our own previous study identified the PI3K pathway as a potential molecular link between HA/CD44 activation and survivin transcription,^42^ and PI3K/AKT increased *KYNU* expression.^12^, ^35^, ^36^ Therefore, CD44‐HA interaction might activate *KYNU* via the PI3K pathway. In conclusion, this review provides several lines of evidence supporting our hypothesis that *KYNU* might be a novel transcriptional target of CD44‐signalling involved in promoting BC cell invasion via NF‐κB and/or PI3K pathways. Ongoing in vitro and in vivo experiments aim to validate *KYNU* as a novel target of CD44‐promoted BC cell invasion and metastasis.

## CONFLICT OF INTEREST

The authors declare that the research was conducted in the absence of any commercial or financial relationships that could be construed as a potential conflict of interest.

## AUTHOR CONTRIBUTION

**Maryam Al‐Mansoob:** Writing‐original draft (lead). **Ishita Gupta:** Writing‐review & editing (equal). **Allal Ouhtit:** Conceptualization (lead); Funding acquisition (lead); Writing‐review & editing (equal). **Radoslaw Stefan Rusyniak:** Editing (equal).

## AUTHOR CONTRIBUTIONS

‘AO: Conceptualization. MAM: writing – original draft. IG and AO: Writing – review and editing. RSR: Editing. AO: funding acquisition. All authors have read and agreed to the published version of the manuscript.’

## Data Availability

Data sharing is not applicable to this article as no new data or datasets were created, generated or analysed in this study.
